# Performance and robustness analysis of V-Tiger PID controller for automatic voltage regulator

**DOI:** 10.1038/s41598-024-58481-1

**Published:** 2024-04-03

**Authors:** Pasala Gopi, S. Venkateswarlu Reddy, Mohit Bajaj, Ievgen Zaitsev, Lukas Prokop

**Affiliations:** 1 Electrical and Electronics Engineering, Annamacharya Institute of Technology and Sciences (Autonomous), Rajampet, India; 2 Department of Electrical Engineering, Graphic Era (Deemed to be University), Dehradun, 248002 India; 3 Hourani Center for Applied Scientific Research, Al-Ahliyya Amman University, Amman, Jordan; 4 Graphic Era Hill University, Dehradun, 248002 India; 5 Applied Science Research Center, Applied Science Private University, Amman, 11937 Jordan; 6 Department of Theoretical Electrical Engineering and Diagnostics of Electrical Equipment, Institute of Electrodynamics, National Academy of Sciences of Ukraine, Peremogy, 56, Kyiv-57, 03680 Ukraine; 7 ENET Centre, VSB—Technical University of Ostrava, 708 00 Ostrava, Czech Republic

**Keywords:** Virtual time response based iterative gain evaluation and re-design, PID controller, Normalized uncertainty, Performance degradation curve, Robust stability margin, AVR, Energy science and technology, Engineering, Mathematics and computing

## Abstract

This paper presents a comprehensive study on the implementation and analysis of PID controllers in an automated voltage regulator (AVR) system. A novel tuning technique, Virtual Time response-based iterative gain evaluation and re-design (V-Tiger), is introduced to iteratively adjust PID gains for optimal control performance. The study begins with the development of a mathematical model for the AVR system and initialization of PID gains using the Pessen Integral Rule. Virtual time-response analysis is then conducted to evaluate system performance, followed by iterative gain adjustments using Particle Swarm Optimization (PSO) within the V-Tiger framework. MATLAB simulations are employed to implement various controllers, including the V-Tiger PID controller, and their performance is compared in terms of transient response, stability, and control signal generation. Robustness analysis is conducted to assess the system's stability under uncertainties, and worst-case gain analysis is performed to quantify robustness. The transient response of the AVR with the proposed PID controller is compared with other heuristic controllers such as the Flower Pollination Algorithm, Teaching–Learning-based Optimization, Pessen Integral Rule, and Zeigler-Nichols methods. By measuring the peak closed-loop gain of the AVR with the controller and adding uncertainty to the AVR's field exciter and amplifier, the robustness of proposed controller is determined. Plotting the performance degradation curves yields robust stability margins and the accompanying maximum uncertainty that the AVR can withstand without compromising its stability or performance. Based on the degradation curves, robust stability margin of the V-Tiger PID controller is estimated at 3.5. The worst-case peak gains are also estimated using the performance degradation curves. Future research directions include exploring novel optimization techniques for further enhancing control performance in various industrial applications.

## Introduction

Many electrical and electronic devices are sensitive to voltage fluctuations. Fluctuations in voltage can damage or reduce the lifespan of equipment connected to the power supply, also affect the stability of the grid, and lead to disruptions in the power supply^1,2^. In power generation plants that are connected to the electrical grid, it is crucial to maintain the voltage within specified limits. The power demand on the generator might fluctuate throughout the day, resulting in voltage spikes or dips as demand suddenly changes^3^. To address these issues, power engineers use a variety of methods and controls to stabilize and manage a power grid's voltage profile^4^.

### Literature on tuning techniques

The most crucial component of the AC generator is the field excitation, which serves as the control mechanism to maintain the voltage level. However, the AVR, which regulates the generator terminal voltage under various operating situations, is the crucial component of the excitation system^5,6^. A PID controller is combined with the AVR to improve the terminal voltage profile of the generator^7,8^. The parameters of a PID controller need to be tuned based on the characteristics of the specific system to achieve optimal performance. The techniques that are used for tuning the gains of the PID controller are classified as classical methods, intelligent techniques, and optimization techniques. Ziegler-Nichols^9,10^ and Cohen-Coon^11,12^ tuning rules are very popularly used conventional techniques to control a wide verity of processes, but large transient response and weak controller robustness are the fundamental drawbacks of the conventional tuning techniques^13,14^. The drawbacks of conventional tuning techniques may be minimized by using intelligent techniques like fuzzy logic^11,15^, artificial neural networks^16,17^, and neural networks^18,19^. Intelligent approaches have practical limits that require expert knowledge, are more difficult to build and tune, and are less interpretable^20,21^. A few optimization algorithms, such as Particle Swarm Optimization^22,23^, Teaching Learning-based Optimization (TLbO), Ant Colony Optimization (ACO), Whale Optimization (WO), Manta Fay Foraging Optimizer (MRFO), Cuckoo Search (CS) Harmony Search (HS), Firefly Algorithm, Flower Pollination Algorithm, Local Unimodal Sampling (LUS) etc., are described in detail^24,25^.

Okou et al.^26^ described stability criteria using the Lyapunov function of the entire power system to generate the non-linear component. Riccati equation with algebraic solutions is used to calculate the gains of the linear component^27,28^. The significant reduction of interconnection signals' influence on voltage and speed dynamics is ensured by these advancements^29,30^. In^31^ the optimal gains of a PID controller in an AVR system are determined using the TLbO approach. Ekinci^32^ presented the salp swarm algorithm (SSA), which offers high-quality adjustment of the ideal PID controller settings for AVR. The FPA is a bio-inspired algorithm that replicates how flower plants naturally conduct pollination. Xin-She Yang recently unveiled it in 2012. Flower plants eventually want to reproduce by pollinating each other^33^. The Adaptive Neuro-Fuzzy Inference System (ANFIS) was created by training the Fuzzy Inference System (FIS). A unique application of ANFIS with a hybrid learning algorithm for the AVR system has been proposed by^34,35^. A new optimization method called Simulink Design Optimization (SDO) is demonstrated in^36^ to evaluate the gains of the PID controller for an AVR. Pachauri^37^ introduced the Water Cycle Algorithm (WCA) to find the PID controller gains optimally. Reconfiguring the distribution system is another way to reduce power loss and enhance the stability of the network^38^. The voltage level at the received end of the network can be maintained constant with the placement of the distributed generators (DGs) at the optimal locations of the network^39^. To effectively stabilize the power system, Meddeb et al. and Shah et al.^40,41^ used power electronic control techniques. The goal of this effort is to investigate how FACTS devices contribute to damping voltage fluctuation in fault conditions. In this article, the Pessen Integral Rule (PIR) and V-Tiger methods for tuning the gains of a PID controller are demonstrated and then compared their performance with other methods such as ZN, PIR, FPA, and TLbO algorithms.

### Research gap and solution

Due to the system dynamics, nonlinear behavior of the AVR components, dynamics of the AC generator and operational constraints, the optimization of AVR is a challenging task^42–44^. The implementation of this innovative method may help to reduce the difficulties associated with AVR optimization^45,46^. V-Tiger is a newly introduced method for adjusting the gains of the PID controller. The advantages of the proposed tuning method are highlighted by comparing its performance with the other PID tuning methods. There are several methods for testing the dynamic system stability in control systems. Among these methods, a pole-zero map is frequently employed because of its ease of use. The Bode plot provides the plant stability in the frequency domain. The plant is more stable the larger the margins^47,48^. The discussion in^36,49^ presents disk stability margins to examine the robustness of the controller and closed-loop stability. In comparison to traditional margin analysis, disk-based margin analysis offers a more robust stability guarantee. In particular research studies, the authors have employed plant parameter uncertainties of ± 25% and ± 50% to assess the robustness of their proposed controllers. However, the maximum uncertainty that a system can withstand without losing its stability is not discussed. This article uses performance degradation curves to illustrate the robustness of the controller and the maximum range of uncertainty that the AVR can withstand without losing its stability.

### Research contributions

The main contributions of the research article are:

1. A novel tuning approach based on system virtual time response is proposed for tuning the gains of the PID controller.
2. Demonstrated that the AVR equipped with the V-Tiger PID controller has a better transient response than the other tuning techniques listed for comparison.
3. Illustrated the performance degradation curves of the AVR with various controllers.
4. The robust stability margins and the corresponding maximum uncertainty, which a system can tolerate without compromising its stability or performance, are measured from the degradation curves.
5. The degradation curves are also used to estimate the worst-case gains at the given uncertainty range.

The rest of this article is structured as follows: The closed-loop transfer function of the AVR is discussed in "Automatic voltage regulator model" section ; the V-Tiger concept and its application to modify the PID gains for the robust AVR is covered in "Evaluating PID controller gains" section; in the section "MATLAB simulation and analysis", the performance and robustness of the AVR utilizing various controllers is discussed. Lastly, this article concludes with research findings.

## Automatic voltage regulator model

**Figure 1:**
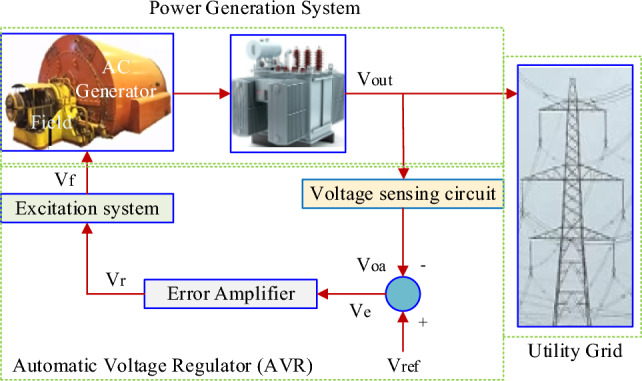
Typical arrangement of power generation with AVR.

An AVR is a control system that regulates the generator voltage to maintain a relatively constant voltage level, despite variations in the load and input conditions^50,51^. The main function of an AVR is to stabilize the generator's output voltage within acceptable limits, ensuring the quality and reliability of electrical power supplied to connected loads^52–54^. Figure 1 shows the schematic diagram of power generation with AVR.

The AVR detects the generator terminal voltage continuously using a voltage sensor. The sensed voltage is compared to a reference voltage (a nominal voltage). The deviation between the sensed voltage (actual output voltage) and the reference voltage is amplified by an error amplifier. The amplified error signal is used to generate a control signal that adjusts the excitation level of the generator's field winding. The control signal is applied to the excitation system, typically through a magnetic amplifier or electronic devices, to adjust the field current of the generator. By controlling the field current, the AVR regulates the magnetic field strength within the generator, affecting the output voltage. The generator's voltage is adjusted to bring it back to the desired level.

### Mathematical model of AVR

A first-order mathematical model of a generator with an AVR can be represented using a simple transfer function. The first-order model typically captures the primary dynamic behavior of the system, specifically focusing on the response of the generator's voltage to changes in the reference voltage or load on the utility grid. In the context of a generator with AVR, this transfer function can be used to model the response of the generator's voltage to changes in the reference voltage. A simplified first-order model for the generator with AVR is

1 $$\begin{aligned} \text{V}_{{\text{out}}} \text{(s)} &= \frac{{\text{K}_{g} }}{{\text{1} + \text{s}\tau_{g} }}\text{V}_{\text{f}} \text{(s)}  \\ \frac{{{\text{V}}_{{{\text{out}}}} ({\text{s}})}}{{{\text{V}}_{{\text{f}}} ({\text{s}})}} &= \frac{{\text{K}}_{g} }{{\text{1} + \text{s}\uptau_{g} }} \end{aligned}$$

Here V_out_(s) = AC generator output voltage; V_f_(s) = Generator field voltage; K_g_ and τ_g_ are the gain and time constants of the AC generator respectively.

In this model, a change in the field voltage V_f_(s) results in a dynamic response of the generator's output voltage V_out_(s). Note that this is a simplified representation, and actual generator systems may involve more complex models that consider additional dynamics, such as the electrical and mechanical dynamics of the generator^55,56^.

Generally, the exciter voltage output is the nonlinear function of the field voltage because of the saturation of the magnetic circuit. Hence there is no simple relationship between the generator output voltage and field exciter voltage. A linearized model of the field exciter in an AVR is obtained by ignoring the saturation effect and other nonlinearities. Practically, the field excitation system is used for adjusting the generator field current to control its output voltage. The simplified first-order model of the field excitation system is

2 $$\begin{aligned} {\text{V}}_{{\text{f}}} ({\text{s}}) &= \frac{{\text{K}_{exc} }}{{\text{1} + \text{s}\uptau_{exc} }}{\text{V}}_{{\text{e}}} ({\text{s}}) \\ \frac{{{\text{V}}{}_{{\text{f}}}({\text{s}})}}{{{\text{V}}_{{\text{e}}} ({\text{s}})}}& = \frac{{\text{K}_{exc} }}{{\text{1} + \text{s}\uptau_{exc} }} \end{aligned}$$

This model suggests that the field voltage V_f_(s) responds to changes in error voltage V_e_(s) (the difference between the reference voltage V_ref_(s) and the actual output voltage V_oa_(s)) with a gain K_exc_ and a time constant τ_exc_. The time constant reflects the speed at which the field voltage adjusts to changes in the reference voltage.

The voltage feedback system is a crucial component in regulating the generator's output voltage^57,58^. The voltage feedback system in an AVR can be formulated as a first-order model by neglecting its saturation effect as an assumption. The first-order transfer function of the voltage feedback system is given below.

3 $$\begin{aligned} {\text{V}}_{{{\text{oa}}}} ({\text{s}}) &= \frac{{\text{K}vf }}{{\text{1} + \text{s}\uptau vf}}{\text{V}}_{\text{out}}({\text{s}}) \\ \frac{{{\text{V}}_{{{\text{oa}}}} ({\text{s}})}}{{{\text{V}}_{{{\text{out}}}} ({\text{s}})}} &= \frac{{\text{K}_{vf}  }}{{\text{1} + \text{s}\uptau_{vf} }} \end{aligned}$$

where V_out_(s) = Generator output voltage; V_oa_(s) = Actual output voltage; K_vf_ and τ_vf_ are the gain and time constants of the voltage feedback sensor. The parameters K_vf_ and τ_vf_ are assumed to be constant, but in reality, these parameters might vary with operating conditions.

The excitation amplifier is an electronic device; it amplifies the voltage error signal before sending it to the excitation system^59^. A mathematical model of the amplifier can be derived by ignoring its nonlinearities and is represented below

4 $$\begin{aligned} {\text{V}}_{{\text{r}}} ({\text{s}}) & = \frac{{\text{K}_{amp} }}{{\text{1} + \text{s}\uptau_{amp} }}\left( {{\text{V}}_{{{\text{ref}}}} ({\text{s}}) - {\text{V}}_{{{\text{oa}}}} ({\text{s}})} \right)  \\  \frac{{{\text{V}}_{{\text{r}}} ({\text{s}})}}{{{\text{V}}_{{\text{e}}} ({\text{s}})}} & = \frac{{\text{K}amp}}{{\text{1} + \text{s}\uptau amp}} \end{aligned}$$

where V_r_ = Amplifier output voltage; V_e_(s) = error between V_ref_(s) and V_ao_(s); K_vf_ and τ_vf_ are the gain and time constants of the voltage feedback sensor. The parameters K_amp_ and τ_amp_ are the gain and time constant of the amplifier respectively.

**Figure 2:**
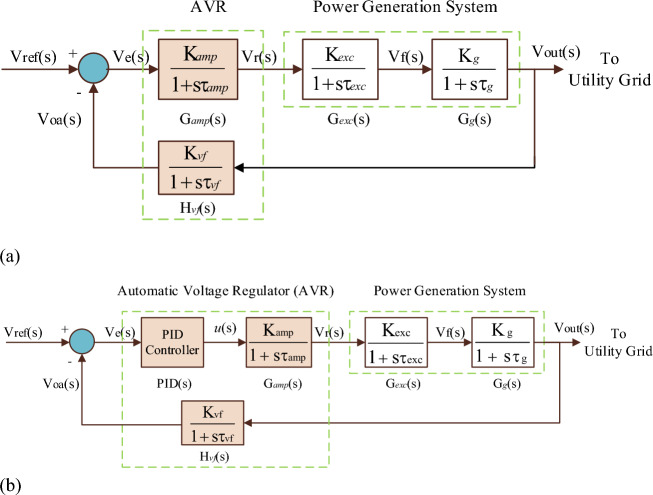
(**a**) Transfer function model of AVR with the power generation system. (**b**) Transfer function model of AVR with PID controller.

Utilizing the above models, the transfer function model of AVR with a power generation system is shown in Fig. 2a.

The closed-loop block diagram in Fig. 2a relating the generator output voltage V_out_(s) to the reference voltage V_ref_(s) is

5 $$\begin{aligned}   \frac{{{\text{V}}_{{{\text{out}}}} ({\text{s}})}}{{{\text{V}}_{{{\text{ref}}}} ({\text{s}})}} &= \frac{{{\text{G}}_{{amp}} ({\text{s}}){\text{G}}_{{{\text{exc}}}} ({\text{s}}){\text{G}}_{g} ({\text{s}})}}{{{\text{1  +  G}}_{{amp}} ({\text{s}}){\text{G}}_{{{\text{exc}}}} ({\text{s}}){\text{G}}_{g} ({\text{s}}){\text{H}}_{{vf}} ({\text{s}})}} \hfill \\   \frac{{{\text{V}}_{{{\text{out}}}} ({\text{s}})}}{{{\text{V}}_{{{\text{ref}}}} ({\text{s}})}} & = \frac{{{\text{K}}_{{amp}} {\text{K}}_{{{\text{exc}}}} {\text{K}}_{g} {\text{K}}_{{vf}} \left( {1 + {\text{s}}\tau _{{vf}} } \right)}}{{\left( {1 + {\text{s}}\tau _{{amp}} } \right)\left( {1 + {\text{s}}\tau _{{exc}} } \right)\left( {1 + {\text{s}}\tau _{g} } \right)\left( {1 + {\text{s}}\tau _{{vf}} } \right) + {\text{K}}_{{amp}} {\text{K}}_{{exc}} {\text{K}}_{g} {\text{K}}_{{vf}} }} \hfill \\  \end{aligned}$$

Equation (5) can be rewritten as

6 $$ {\text{V}}_{{{\text{out}}}} ({\text{s}}) = \frac{{\text{K}_{{amp }} \text{K}_{exc} \text{ K}_{g} \text{K}_{vf} \text{ (1} + \text{s}\uptau_{vf} \text{)}}}{{\text{(1} + \text{s}\uptau_{amp} \text{) (1} + \text{s}\uptau_{exc} \text{) (1} + \text{s}\uptau_{g} \text{) (1} + \text{s}\uptau_{vf} \text{)} + \text{K}_{amp} \text{ K}_{exc} \text{ K}_{g} \text{ K}_{vf} }}{\text{V}}_{{{\text{ref}}}} ({\text{s}}) $$

For a unit step reference voltage V_ref_(s) = $\frac{1}{\text{s}}$, the steady-state error

7 $$ {\text{V}}_{{{\text{ess}}}} \left( {\text{s}} \right) = \left| {{\text{V}}_{{{\text{ref}}}} {\text{(s) }}} \right| - \mathop {\lim }\limits_{s \to 0} {\text{sV}}_{{{\text{out}}}} \left( {\text{s}} \right) $$

The steady-state error defined in (7) can be eliminated by adding the controller to AVR. The controller's role is not just about eliminating steady-state errors but also about maintaining voltage regulation, ensuring system stability, responding to dynamic changes, and preventing voltage violations in different operating conditions^60,61^. In this research article, a PID controller is used. A PID controller is a widely used feedback control system in various industrial applications. It regulates the response of the plant by adjusting the input based on the error generated by the required set-point and the actual response.

The control signal *u(t)* from the PID controller is mathematically represented as^62^.

8 $$ u(t) = \text{K}_{\text{p}}  e(t) + \text{K}_{\text{i}} \int\limits_{\text{0}}^{\text{t}} {e\left( \uptau \right) d\uptau + \text{K}_{\text{d}} \frac{de(t)}{{dt}}} $$

where *e(t)* is the error at time *t*, K_p_ is the proportional gain, K_i_ is the integral gain and K_d_ is the derivative gain. Figure 2b shows the transfer function model of AVR with PID controller.

The closed-loop transfer function of block diagram in Fig. 2b relating the generator output voltage V_out_(s) to the reference voltage V_ref_(s) is

9 $$\begin{aligned}   \frac{{{\text{V}}_{{{\text{out}}}} ({\text{s}})}}{{{\text{V}}_{{{\text{ref}}}} ({\text{s}})}}  & = \frac{{{\text{PID}}({\text{s}}){\text{G}}_{{amp}} ({\text{s}}){\text{G}}_{{exc}} ({\text{s}}){\text{G}}_{g} ({\text{s}})}}{{{\text{1 + PID}}({\text{s}}){\text{G}}_{{amp}} ({\text{s}}){\text{G}}_{{exc}} ({\text{s}}){\text{G}}_{g} ({\text{s}}){\text{H}}_{{vf}} ({\text{s}})}} \hfill \\     & = \frac{{\left( {{\text{s}}^{2} {\text{K}}_{{\text{d}}}  + {\text{sK}}_{{\text{p}}}  + {\text{K}}_{{\text{i}}} } \right){\text{K}}_{{amp}} {\text{K}}_{{exc}} {\text{K}}_{g} \left( {1 + {\text{s}}\tau _{{vf}} } \right)}}{{{\text{s}}\left( {1 + {\text{s}}\tau _{{amp}} } \right)\left( {1 + {\text{s}}\tau _{{exc}} } \right)\left( {1 + {\text{s}}\tau _{g} } \right)\left( {1 + {\text{s}}\tau _{{amp}} } \right) + \left( {{\text{s}}^{2} {\text{K}}_{{\text{d}}}  + {\text{sK}}_{{\text{p}}}  + {\text{K}}_{{\text{i}}} } \right){\text{K}}_{{amp}} {\text{K}}_{{exc}} {\text{K}}_{g} {\text{K}}_{{vf}} }} \hfill \\  \end{aligned}$$

The nominal values and proposed uncertainty of the AVR system, presented in Fig. 2a, are given in Table 1^36,47^. By utilizing these nominal parameters, the closed-loop transfer function of the AVR is

10 $$ \frac{{{\text{V}}_{{{\text{out}}}} ({\text{s}})}}{{{\text{V}}_{{{\text{ref}}}} ({\text{s}})}} = \frac{{\text{0.1s} + \text{10}}}{{\text{0.0004 s}^{\text{4}} + \text{0.0454 s}^{\text{3}} + \text{0.555 s}^{\text{2}} + 1.51\text{s} + 11}} $$

**Table 1:** Nominal values and proposed uncertainty of AVR components.

Component	Nominal values		Proposed uncertainty	
	Gain constant	Time constant	Gain constant	Time constant
AC generator	1.0	1.0	No uncertainty	
Field exciter	1.0	0.4	50%	50%
Voltage feedback	1.0	0.01	No uncertainty	
Amplifier	10.0	0.1	50%	50%

Also, the closed-loop transfer function of the AVR including the PID controller is given as

11 $$\frac{{{\text{V}}_{{{\text{out}}}} ({\text{s}})}}{{{\text{V}}_{{{\text{ref}}}} ({\text{s}})}} = \frac{{0.1{\text{K}}_{{\text{d}}} {\text{s}}^{3}  + \left( {0.1{\text{K}}_{{\text{p}}}  + 10{\text{K}}_{{\text{d}}} } \right){\text{s}}^{2}  + \left( {0.1{\text{K}}_{{\text{i}}}  + 10{\text{K}}_{{\text{p}}} } \right){\text{s}} + 10{\text{K}}_{{\text{i}}} }}{{0.0004{\text{s}}^{5}  + 0.0454{\text{s}}^{4}  + 0.555{\text{s}}^{3}  + \left( {1.51 + 10{\text{K}}_{{\text{d}}} } \right){\text{s}}^{2}  + \left( {1 + 10{\text{K}}_{{\text{p}}} } \right){\text{s}} + 10{\text{K}}_{{\text{i}}} }} $$

## Evaluating PID controller gains

Tuning a PID controller is crucial for the proper functioning and performance of a control system^63,64^. Proper tuning ensures that the controller effectively regulates the system, minimizing errors and achieving the desired dynamic response^65^. This article proposes the Pessen Integral Rule (PIR) and Virtual Time response-based iterative gain evaluation and re-designs (V-Tiger) methods to tune the PID controller. The results of the proposed controller are compared with FPA, TLbO, PIR, and ZN-based PID controllers.

### Pessen Integral Rule

**Figure 3:**
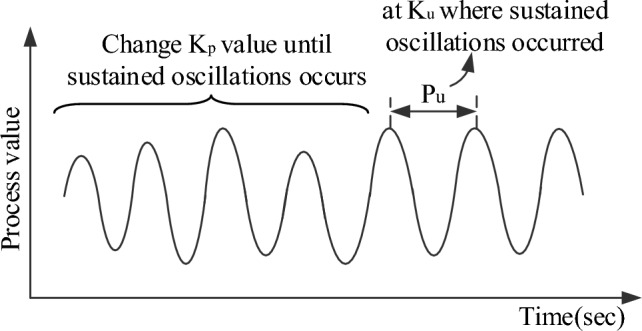
Plant response with step-response experiment.
  Figure 3 states that the ultimate period (P_u_) is the interval of time between two successive peaks, and the ultimate gain (K_u_) is the proportional control gain at which persistent oscillations occur.
Step-II: *Calculate the integral time constant (T*_*i*_*)*
  Calculate the integral and derivative time constants by applying the Pessen integral rule i.e. integral time constant (T_i_) = P_*u*_/4 and derivate time constant T_d_ = 0.15P_u_.
Step-III: *Set PID controller parameters*
  $${\text{The standard form of PID controller is u}}\left( {\text{s}} \right){\mkern 1mu}  = {\text{K}}_{{\text{p}}} {\mkern 1mu} \left( {1{\mkern 1mu}  + {\mkern 1mu} \frac{1}{{{\text{sT}}_{{\text{i}}} }}{\mkern 1mu}  + {\text{ sT}}_{{\text{d}}} } \right) = {\text{K}}_{{\text{p}}}  + {\mkern 1mu} \frac{1}{{\text{s}}}{\mkern 1mu} {\text{K}}_{{\text{i}}}  + {\text{ sK}}_{{\text{d}}}.$$
  Set the PID controller gains by using the Pessen Integral Rule as follows
  K_p_ = 0.7K_*u*_; K_i_ = $$\frac{{{\text{K}}_{{\text{p}}} }}{{{\text{T}}_{{\text{i}}} }}$$ = 0.175 $$\frac{{{\text{K}}_{u} }}{{{\text{P}}_{u} }}$$ and K_d_ = K_p_T_d_ = 0.105K_*u*_P_*u*_*.*

The Pessen Integral Rule (PIR) is another tuning method for PID controllers. It was proposed by Leonard Pessen and is a modification of the ZN tuning method. This method emphasizes the integral action by setting the integral time constant to one-fourth of the ultimate period obtained from the Ziegler-Nichols method^66,67^. The Pessen Integral Rule aims to improve the transient response of the system by giving more weight to the integral term. Here are the steps to apply the Pessen Integral Rule:

Step-I: *Perform step response experiment*
  Using proportional control alone, do a step-response experiment to obtain the plant's ultimate gain (K_u_) and ultimate period (P_u_). Adjust the proportional gain (K_p_) until the response exhibits the persistent oscillations as seen in Fig. 3.

### Virtual time response based iterative gain evaluation and re-design (V-Tiger)

The V-Tiger approach to tune a PID (Proportional-Integral-Derivative) controller involves virtual time-response analysis and iterative gain adjustment to optimize the PID parameters for a given system^68,69^. Using this tuning procedure, the frequency characteristics of the controller are combined with the computed frequency components of the controlled object's input and output. The closed-loop system time response is obtained by first calculating the response in the frequency domain, and then using the inverse Fourier transformation. With this ideal, one-shot experimental data is used to compute the controlled plant's time response. The time response obtained by this method is known as Virtual time response^70^.

Let the controlled plant G(z) be a linear time invariant discrete-time SISO system. The input and output time series of the one-shot experimental data of G(z) are, respectively, u_0_(k) and y_0_(k). In this case, k is the sample number (= 1, 2, 3,…, n) and z is the shift operator. Let the time series of the reference signal (input), disturbance signal (output), and disturbance signal (input) to the closed-loop system be denoted by ref(k), δ(k), and δu(k). The discrete fourier transformations of u_0_(k), y_0_(k), ref(k), δ(k), and δ_u_(k) are u_0_(jω), y_0_(jω), ref(jω), δ(jω), and δ_u_(jω), respectively. The angular velocity (rad/sec) is denoted by ω, which may be expressed mathematically as follows in terms of sampling time (t_s_): ω = 0, 2π/nt_s_, 4π/nt_s_ . . . (n − 1)2π/nt_s_. The following presumptions were made to use this method:

i. The input time series of one-shot experimental data u_0_(jω_a_) ≠ 0 at a given angular velocity ω_a_ if ref(jω_a_), δ(jω_a_), and δ_u_(jω_a_) are not 0, and vice versa.
ii. The closed-loop system should be stabilized by the controller C_PID_. If controller C_PID_(jω) ≠ 0, then there is no stability boundary pole for any value of ω.
iii. The inverse of plant G^-1^(jω_a_) has a stability boundary pole at a given angular velocity ω_a_ if $\text{C}_{\text{PID}}^{ - 1} (j\upomega_{\text{a}} )$ ≠ 0, and vice versa.
iv. The initial and final values of step response data u_00_ and y_00_ are considered as steady state.

#### Basic procedure

**Figure 4:**
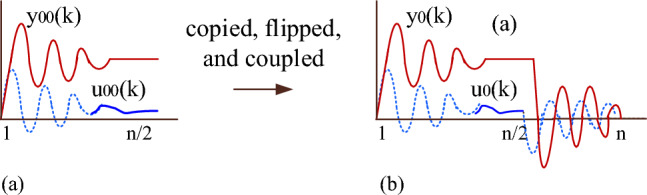
(**a**) Step response. (**b**) Cyclic response.

The under-dampened step response in Fig. 4a is copied, flipped, and coupled to the original step response as shown in Fig. 4b. In terms of u_00_(k) and y_00_(k), the input and output time series data (u_0_(k) and y_0_(k)) of G(z) are represented as

12 $$ {\text{u}}_{0} \left( {\text{k}} \right) \, = \left\{ {\begin{array}{ll} {{\text{u}}_{00} \left( {\text{k}} \right),} & \quad {{\text{k }} = { 1},{ 2}, \, \ldots {\text{n}}/{2}.} \\ { - {\text{ u}}_{00} \left( {{\text{k}} - \frac{{\text{n}}}{2}} \right) + {\text{ u}}_{00} \left( {1} \right) \, + {\text{ u}}_{00} \left( {\frac{{\text{n}}}{2}} \right),} & \quad {{\text{k}} = \frac{{\text{n}}}{2} + 1,\;\frac{{\text{n}}}{2} + 2, \ldots {\text{n}}.} \\ \end{array} } \right. $$

13 $$ {\text{y}}_{0} \left( {\text{k}} \right) \, = \left\{ {\begin{array}{ll} {{\text{y}}_{00} \left( {\text{k}} \right),} & \quad {{\text{k }} = { 1},{ 2}, \, \ldots {\text{n}}/{2}.} \\ { - {\text{ y}}_{00} \left( {{\text{k}} - \frac{{\text{n}}}{2}} \right) + {\text{ y}}_{00} \left( {1} \right) \, + {\text{ y}}_{00} \left( {\frac{{\text{n}}}{2}} \right),} & \quad {{\text{k}} = \frac{{\text{n}}}{2} + 1,\;\frac{{\text{n}}}{2} + 2, \ldots {\text{n}}.} \\ \end{array} } \right. $$

Since the frequency response has only a steady-state response, the discrete-time frequency function of plant G and controller C_PID_ can be written, respectively, as G(jω), C_PID_(jω). Now the time series of output y_0_(jω) can be represented as

14 $$ {\text{y}}_{0} \left( {{\text{j}}\upomega } \right) \, = {\text{ G}}\left( {{\text{j}}\upomega } \right){\text{ u}}_{0} \left( {{\text{j}}\upomega } \right) $$

**Figure 5:**
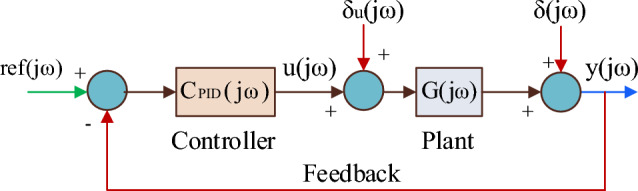
Closed-loop plant with controller.

Figure 5 depicts the closed-loop plant when the controller (whose control performance is to be evaluated) is introduced.

Considering the disturbances δ(jω) and δ_u_(jω), Fig. 5 holds

15 $$ {\text{y}}\left( {{\text{j}}\upomega } \right) \, = \frac{{{\text{G}}({\text{j}}\upomega ){\text{ C}}_{{{\text{PID}}}} ({\text{j}}\upomega )}}{{1 + {\text{G}}({\text{j}}\upomega ){\text{ C}}_{{{\text{PID}}}} ({\text{j}}\upomega )}}{\text{ref}}({\text{j}}\upomega ) + \frac{1}{{1 + {\text{G}}({\text{j}}\upomega ){\text{ C}}_{{{\text{PID}}}} ({\text{j}}\upomega )}}\updelta ({\text{j}}\upomega ) + \frac{{{\text{G}}({\text{j}}\upomega )}}{{1 + {\text{G}}({\text{j}}\upomega ){\text{ C}}_{{{\text{PID}}}} ({\text{j}}\upomega )}}\updelta {\text{u}}({\text{j}}\upomega ) $$

from (14), (15) and multiplying the results on both numerator and denominator with $\text{C}_{\text{PID}}^{ - 1} ({\text{j}}\upomega )$ u_0_(jω)

16 $$ {\text{y}}\left( {{\text{j}}\upomega } \right) \, = \frac{{{\text{y}}_{0} ({\text{j}}\upomega ){\text{ref}}({\text{j}}\upomega )}}{{{\text{C}}_{{{\text{PID}}}}^{ - 1} ({\text{j}}\upomega ){\text{u}}_{0} ({\text{j}}\upomega ) + {\text{y}}_{0} ({\text{j}}\upomega )}} + \frac{{{\text{C}}_{{{\text{PID}}}}^{ - 1} ({\text{j}}\upomega ){\text{u}}_{0} ({\text{j}}\upomega )\updelta ({\text{j}}\upomega )}}{{{\text{C}}_{{{\text{PID}}}}^{ - 1} ({\text{j}}\upomega ){\text{u}}_{0} ({\text{j}}\upomega ) + {\text{y}}0({\text{j}}\upomega )}} + \frac{{{\text{C}}_{{{\text{PID}}}}^{ - 1} ({\text{j}}\upomega ){\text{u}}_{0} ({\text{j}}\upomega )\updelta u({\text{j}}\upomega )}}{{{\text{C}}_{{{\text{PID}}}}^{ - 1} ({\text{j}}\upomega ){\text{u}}_{0} ({\text{j}}\upomega ) + {\text{y}}0({\text{j}}\upomega )}} $$

Assume ref_1_(jω) = $\text{C}_{\text{PID}}^{ - 1} ({\text{j}}\upomega )\text{u}_{0}(\text{j}\upomega ) + \text{y}_{0}(\text{j}\upomega ),$ the (16) can be re-written as

17 $$ {\text{y}}\left( {{\text{j}}\upomega } \right) \, = \frac{{{\text{ref}}(j\upomega )}}{{{\text{ref}}_{1} (j\upomega )}}{\text{y}}0({\text{j}}\upomega ) + \frac{\updelta (j\upomega )}{{{\text{ref}}_{1} (j\upomega )}}{\text{C}}_{{{\text{PID}}}}^{ - 1} ({\text{j}}\upomega ){\text{u}}_{0} ({\text{j}}\upomega ) + \frac{{\updelta_{{\text{u}}} ({\text{j}}\upomega )}}{{{\text{ref}}_{1} ({\text{j}}\upomega )}}{\text{C}}_{{{\text{PID}}}}^{ - 1} ({\text{j}}\upomega ){\text{u}}_{0} ({\text{j}}\upomega ) $$

Also from Fig. 5,

18 $$ {\text{u}}\left( {{\text{j}}\upomega } \right) \, = {\text{ C}}_{{{\text{PID}}}} \left( {{\text{j}}\upomega } \right)\left( {{\text{ref}}\left( {{\text{j}}\upomega } \right) \, {-}{\text{ y}}\left( {{\text{j}}\upomega } \right)} \right) $$

**Figure 6:**
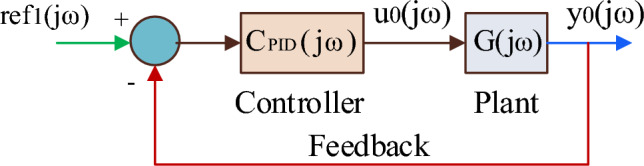
Closed-loop plant with ref(jω) and δ(jω) = δ_u_(jω) = 0.

Assuming δ = δ_u_ = 0 and giving ref_1_(jω) as input to the closed-loop plant, Fig. 5 can be re-drawn as shown in Fig. 6.

At a certain value of angular velocity (ω_a_), when ref_1_(jω_a_) = 0, from Fig. 6

19 $$\begin{aligned} {\text{y}}_{0} \left( {{\text{j}}\upomega_{{\text{a}}} } \right) & = \frac{{{\text{G}}({\text{j}}\upomega_{{\text{a}}} ){\text{ C}}_{{{\text{PID}}}} ({\text{j}}\upomega_{{\text{a}}} )}}{{1 + {\text{G}}({\text{j}}\upomega_{{\text{a}}} ){\text{C}}_{{{\text{PID}}}} ({\text{j}}\upomega_{{\text{a}}} )}}{\text{ref}}_{{1}} ({\text{j}}\upomega_{{\text{a}}} )  \\ {\text{y}}_{0} \left( {{\text{j}}\upomega_{{\text{a}}} } \right) & = \frac{1}{{1 + \frac{1}{{{\text{G}}({\text{j}}\upomega_{a} ){\text{ C}}_{{{\text{PID}}}} ({\text{j}}\upomega_{a} )}}}}0 \end{aligned}$$

20 $$\begin{aligned} {\text{u}}_{0} \left( {{\text{j}}\upomega_{{\text{a}}} } \right) & = \frac{{{\text{C}}_{{{\text{PID}}}} ({\text{j}}\upomega_{{\text{a}}} )}}{{1 + {\text{G}}({\text{j}}\upomega_{{\text{a}}} ){\text{ C}}_{{{\text{PID}}}} ({\text{j}}\upomega_{{\text{a}}} )}}{\text{ref}}_{{1}} ({\text{j}}\upomega_{{\text{a}}} )  \\ {\text{u}}_{0} \left( {{\text{j}}\upomega_{{\text{a}}} } \right) & = \frac{1}{{{\text{G}}({\text{j}}\upomega_{{\text{a}}} ) + \frac{1}{{{\text{ C}}_{{{\text{PID}}}} ({\text{j}}\upomega_{{\text{a}}} )}}}}{\text{ref}}_{1} ({\text{j}}\upomega_{{\text{a}}} ) = \frac{1}{{{\text{G}}({\text{j}}\upomega_{{\text{a}}} ) + {\text{C}}_{{{\text{PID}}}}^{ - 1} ({\text{j}}\upomega_{{\text{a}}} )}}0 \end{aligned}$$

In (19), if $1 + \frac{1}{\text{G}(\text{j}\upomega \text{a}) \, \text{CPID}(\text{j}\upomega a)}$ ≠ 0, y_0_(jω_a_) becomes zero. In (20), if ${\text{G}}({\text{j}}\upomega_{{\text{a}}} ) + {\text{C}}_{{{\text{PID}}}}^{ - 1} ({\text{j}}\upomega_{{\text{a}}} )$ ≠ 0, u_0_(jω_a_) = 0. When G(jω_a_)C_PID_(jω_a_) = − 1 or G(jω_a_) = 0 or ${\text{C}}_{{{\text{PID}}}}^{ - 1} ({\text{j}}\upomega_{{\text{a}}} )$ = 0, $\text{G}(\text{j}\upomega_{\text{a}} ) + \text{C}_{\text{PID}}^{ - 1} (\text{j}\upomega \text{a})$ = 0. From (20), with the assumption—iii, when G(jω_a_) = 0, ${\text{C}}_{{{\text{PID}}}}^{ - 1} ({\text{j}}\upomega_{{\text{a}}} )$ is non-zero, hence u_0_(jω_a_) = 0. A similar argument can be made, for Fig. 5, when ref(jω_a_) and δ(jω_a_) = δ_u_(jω_a_) = 0. From (15), at ω_a_

$$ {\text{y}}\left( {{\text{j}}\upomega_{{\text{a}}} } \right) \, = \frac{1}{{1 + 1/\left( {{\text{G}}({\text{j}}\upomega_{{\text{a}}} ){\text{ C}}_{{{\text{PID}}}} ({\text{j}}\upomega_{{\text{a}}} )} \right)}}0 + \frac{1}{{1 + {\text{G}}({\text{j}}\upomega_{{\text{a}}} ){\text{ C}}_{{{\text{PID}}}} ({\text{j}}\upomega_{{\text{a}}} )}}0 + \frac{1}{{{\text{G}}^{ - 1} ({\text{j}}\upomega_{{\text{a}}} ) + {\text{C}}_{{{\text{PID}}}}^{{}} ({\text{j}}\upomega_{{\text{a}}} )}}0 $$

With assumption (ii), the 1st and 2nd terms become ‘0’. When ${\text{G}}^{ - 1} ({\text{j}}\upomega_{\text{a}} ) + {\text{C}}_{\text{PID}}^{{}} ({\text{j}}\upomega \text{a})$ = 0, the 3rd term is ‘0’. The term ${\text{G}}^{ - 1} ({\text{j}}\upomega_{\text{a}} ) + {\text{C}}_{\text{PID}}^{{}} ({\text{j}}\upomega {\text{a}})$ = 0 only when ${\text{G}}({\text{j}}\upomega_{\text{a}} ) \, {\text{CPID}}({\text{j}}\upomega_{\text{a}} )$ = − 1 or ${\text{G}}^{ - 1} ({\text{j}}\upomega_{\text{a}} ) \, =$ ${\text{CPID}}({\text{j}}\upomega_{\text{a}} )$ = 0. Therefore, y(jω_a_) = 0. Putting y(jω_a_) and ref(jω_a_) as 0 in (18), G(jω_a_) = C_PID_(jω_a_)0.0 or G(jω_a_) = 0 when ${\text{C}}_{{{\text{PID}}}}^{{ - 1}} \left( {{\text{j}}\upomega _{{\text{a}}} } \right)$ ≠ 0. Now by putting y(jω_a_) = 0 in y(jω_a_) = G(jω_a_)u(jω_a_), u(jω_a_) becomes zero. The time responses y and u are the inverse discrete Fourier transformations of y(jω) and u(jω) with the assumptions (i), (ii), and (iii).

### Implementation of V-Tiger for controller gains

Before applying V-Tiger, model the system to be controlling. Develop a mathematical model that represents the dynamics of the plant under control. Initialize the PID controller with initial gain values. These initial values are based on any heuristic methods or values commonly used in similar systems. In this work, the initial PID gains are based on the Pessen Integral Rule. Perform the virtual time-response analysis using the modeled system and the initial PID controller settings. Simulate the closed-loop system and observe the system's response. Now quantify the performance metrics such as overshoot, settling time, and stability margin from the virtual time-response analysis. These metrics will be used to evaluate the performance of the system under the current PID controller settings. Based on this virtual time-response analysis, adjust the PID gains iteratively. To adjust the PID gains iteratively, Particle Swarm Optimization (PSO) is used in this research. Repeat the virtual time-response analysis with the updated PID gains. Measure the performance metrics again and assess whether the changes result in improved control performance. Continue the iterative process of adjusting PID gains, performing virtual experiments, and evaluating system performance until the desired control performance is achieved. The objective function is defined in (21) with the values of overshoot and stability margins as constraints.

21 $$ {\text{Objective function}},\;\;min\left( {\text{J}} \right) \, = \text{t}\int {\left| {\text{V}_{{\text{ref}}} - \text{V}_{{\text{oa}}} } \right|} +  \upomega_{{1}} {\text{t}}_{{\text{s}}} + \, \upomega_{{2}} \frac{{{\text{Os}}}}{100} $$

**Figure 7:**
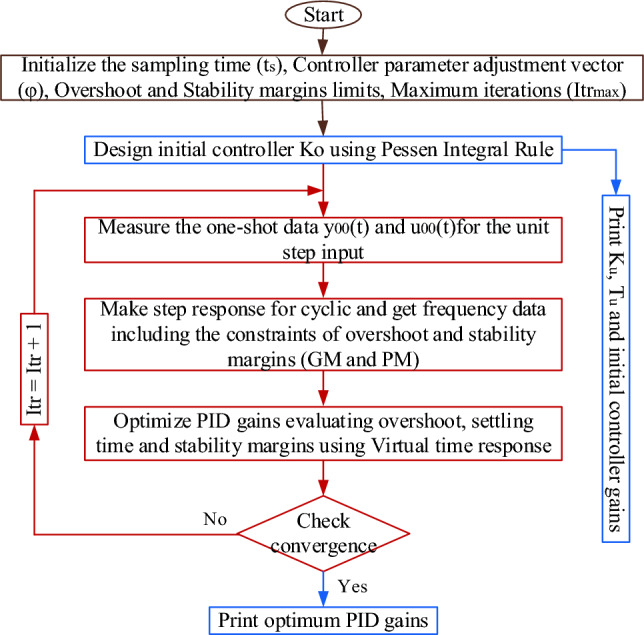
Flowchart of V-Tiger.

To adjust the controller's behavior to fulfill the performance needs, satisfy the constraints, and enhance the robustness of the AVR, here are two weighting factors, ω_1_ and ω_2_, selected as 4 and 2, respectively. Os = Overshoot. Figure 7 shows the flow chart of the V-Tiger.

## MATLAB simulation and analysis

The suggested controllers are implemented in an AVR system that is simulated using 2021b MATLAB/Simulink. The nominal parameters and their associated uncertainties are shown in Table 1. By implementing the ZN ultimate sensitivity method to (10), the critical gain K_*u*_ and the ultimate period P_*u*_ are identified as 1.6053 and 1.121 s respectively. Using the Pessen Integral Rule (PIR), the PID controller gains are K_p_ = 1.1235, K_i_ = 2.509, and K_d_ = 0.189. These gains are considered as initial PID controller gains for obtaining the optimum PID controller using Virtual Time response-based iterative gain evaluation and re-designs (V-Tiger) with the constraints (i) Overshoot ≤ 10%, (ii) Stability margins GM > 3 dB and PM  >  20°. The search range for controller gains using V-Tiger is from zero to twice the PID gains of the Pessen Integral Rule. After carrying 30 simulation runs, the optimal gains of the V-Tiger PID controller are identified as K_p_ = 0.8415, K_i_ = 0.6163, and K_d_ = 0.2838.

### Transient response analysis

**Figure 8:**
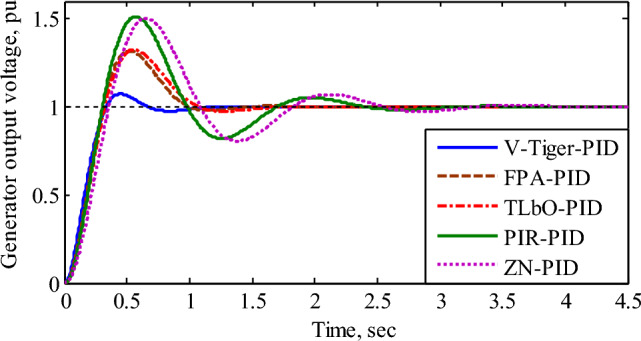
Transient response of the generator output voltage.

The transient response of the AVR without a controller possesses a percentage overshoot of 50.53%, settling time of 6.99 s with steady-state error of 0.0909 pu^36^. Figure 8 displays, for various controllers, the transient response of the generator output voltage (pu). Table 2 lists the step response specifications, stability performance, and closed loop poles with the damping ratio of the AVR utilizing various controllers.

**Table 2:** Comparison of time response, stability performance, and closed-loop poles with damping ratio for different controllers.

Controller type	Time response specifications			Stability performance		Closed loop poles and damping ratio (ξ)
	T_r_ (s)	T_s_ (s)	%Os	GM (dB)	PM (Deg)	
No controller	0.437	6.99	50.53	4.62@5.77	16.1@4.4	P_1_ = − 100, P_2_ = − 12.5 with ξ = 1 P_3&4_ = − 0.51 ± *i* 4.66 with ξ = 0.11
V-Tiger-PID	0.219	0.931	7.32	23@30.2	60@5.91	P_1_ = − 100.78, with ξ = 1 P_2&3_ = − 1.40 ± *i* 0.25 with ξ = 0.985 P_4&5_ = − 4.96 ± *i* 7.15 with ξ = 0. 57
FPA-PID	0.215	0.10	29.5	18@20.1	42.1@5.33	P_1_ = − 100.59, with ξ = 1 P_2&3_ = − 3.01 ± *i* 0.74 with ξ = 0.971 P_4&5_ = − 3.45 ± *i* 5.06 with ξ = 0. 563
TLbO-PID	0.226	1.49	30.4	18.9@20	42@5.01	P_1_ = − 100.54, with ξ = 1 P_2&3_ = − 3.14 ± *i* 1.37 with ξ = 0.916 P_4&5_ = − 3.34 ± *i* 4.47 with ξ = 0. 598
PIR-PID	0.202	2.28	51.20	18.2@18.7	29@4.94	P_1_ = − 100.51, with ξ = 1 P_2&3_ = − 1.55 ± *i* 4.28 with ξ = 0.341 P_4&5_ = − 4.94 ± *i* 2.39 with ξ = 0.9
ZN-PID	0.236	3.04	50.30	19.5@17.1	27.6@4.4	P_1_ = − 100.86, with ξ = 1 P_2&3_ = − 5.06 ± *i* 7.53 with ξ = 0.341 P_4&5_ = − 12.6 ± *i* 1.9 with ξ = 0.558

T_r_ = Rise time; T_s_ = Settling time; %Os = Percentage Overshoot; GM = Gain margin at rad/sec; PM = Phase margin at rad/sec.

**Figure 9:**
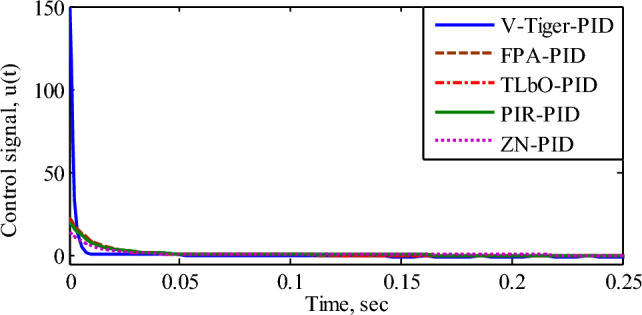
Comparison of control signals by different PID controllers.

**Figure 10:**
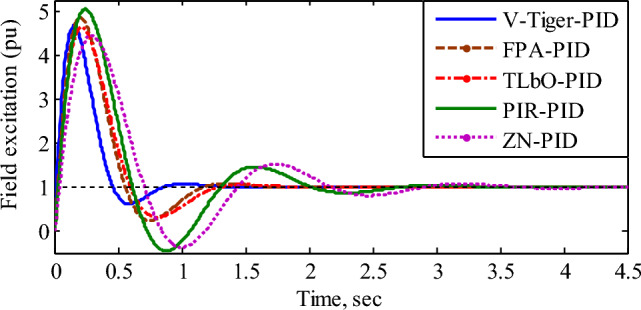
Field excitation (pu) for various PID controllers.

According to Fig. 8 and Table 2, the AVR using the V-Tiger PID controller possesses superior transient response, more stable margin range, and damping ratio than the other controllers. Hence the AVR with V-Tiger PID controller is more stable and exhibits good performance. Figure 9 compares the control signals generated by the various PID controllers. Figure 10 illustrates how different PID controllers provide field excitation to AC generator.

### Robustness analysis

**Figure 11:**
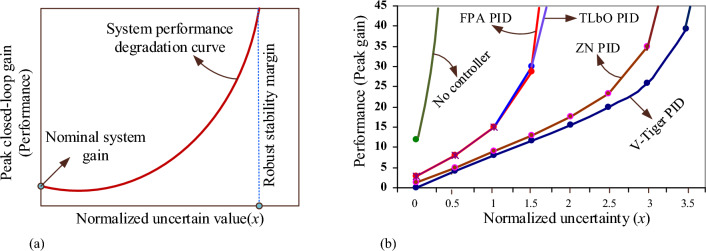
(**a**) System performance degradation curve. (**b**) AVR performance degradation curve with controllers.

Robustness analysis is a crucial aspect of system design and control engineering. It involves examining how well a system can maintain stability and desired performance levels in the presence of uncertainties or variations in its parameters. The goal is to identify the maximum amount of uncertainty that a system can tolerate without compromising its stability or performance. The trade-off curve, as shown in Fig. 11a, provides a visual representation of the relationship between the normalized amount of uncertainty in the system and its performance, specifically measured by the peak gain of the closed-loop transfer function using the Bode plot. In Fig. 11a, the value of normalized uncertainty (*x*) = 1 corresponds to the uncertainty ranges specified in the model. This is considered the nominal level of uncertainty. When *x* = 2, it represents a system with twice as much uncertainty compared to the nominal system. *x* = 0 corresponds to the nominal system. Performance is represented on the y-axis by the peak gain of a closed-loop transfer function. From the graph, it can be inferred that the plant moves into an unstable zone when the normalized uncertainty rises with an increase in peak gain.

The performance degradation curves of the AVR with various controllers are shown in Fig. 11(b). This graph indicates that in the absence of a controller, the AVR system becomes unstable for even small amounts of system uncertainty. The AVR with V-Tiger PID controller is more stable than the FPA PID, TLbO PID, and ZN PID controllers in terms of system uncertainty as well. The peak gains and time delays of the AVR system with various PID controllers for the normalized uncertainty range of the amplifier and exciter are listed in Table 3.

**Table 3:** Peak gains and time delays of AVR for the normalized uncertainty of amplifier and exciter.

Normalized uncertainty	V-Tiger-PID		FPA-PID		TLbO-PID		PIR-PID		ZN-PID	
	K_Pg_	t_d_	K_Pg_	t_d_	K_Pg_	t_d_	K_Pg_	t_d_	K_Pg_	t_d_
*x* = 0	0.025	0.405	2.78	0.197	2.84	0.213	6.24	0.155	1.40	0.246
*x* = 0.5	4.16	0.0938	8.07	0.0694	8.10	0.0742	13.70	0.050	4.91	0.0806
*x* = 1	7.95	0.0532	15	0.0286	15.1	0.0303	47	0.0085	8.98	0.0453
*x* = 1.5	11.6	0.0333	28.6	0.0066	30.1	0.0066	Unstable		13	0.0275
*x* = 2	15.4	0.0211	Unstable		Unstable		Unstable		17.6	0.0164
*x* = 2.5	19.9	0.0126	Unstable		Unstable		Unstable		23.4	0.0087
*x* = 3	25.9	0.0063	Unstable		Unstable		Unstable		34.9	0.0029
*x* = 3.5	39.2	0.0015	Unstable		Unstable		Unstable		Unstable	

K_Pg_ = Peak gain (Closed loop), dB; t_d_ = Delay margin, sec.

**Figure 12:**
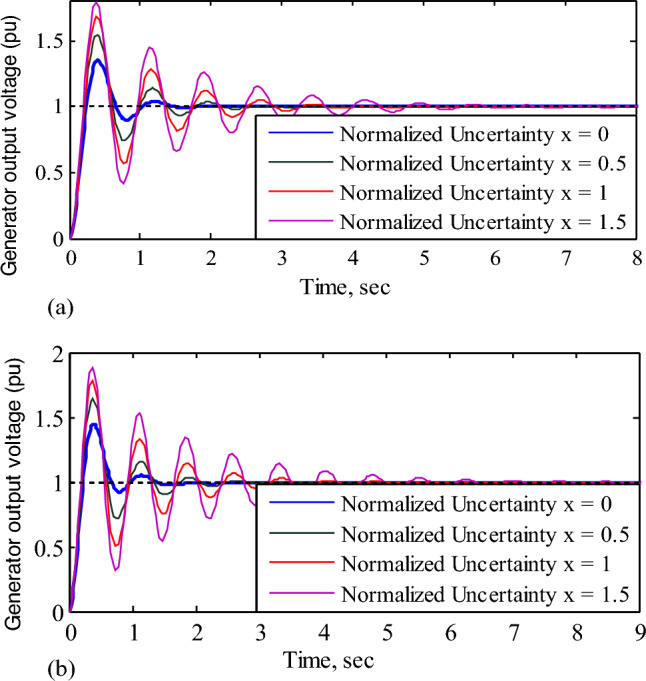
(**a**) Response of the AVR with V-Tiger-PID controller for normalized uncertainty. (**b**) Response of the AVR with ZN-PID controller for normalized uncertainty.

According to Table 3, when the uncertainty of the amplifier and exciter are set to their nominal values (*x* = 0), the nominal system gain (peak) with V-Tiger-PID controller is around 0.025dB. The peak gain of the AVR with the V-Tiger PID controller is infinite at normalized uncertainty (*x*) of amplifier and exciter of AVR is 3.5 i.e. the AVR system becomes unstable when the uncertainty range of the amplifier and exciter of AVR is beyond 3.5 times the specified uncertainty of the AVR model. Therefore, the robust stability margin of the AVR with the V-Tiger PID controller is 3.5. A robust stability margin means it is the maximum value of uncertainty that the system can withstand and remains stable. Similarly, for the AVR using FPA-PID, TLbO-PID, PIR-PID, and ZN-PID controllers, respectively, the peak gains of the AVR become infinite beyond the normalized uncertainty *x* = 1.5, *x* = 1.5, *x* = 1, and *x* = 3 and the robust stability margins are identified as 1.5, 1.5, 1 and 3. Figure 12a and b show the AVR responses for different normalized uncertainty levels using V-Tiger and ZN PID controllers, respectively.

Table 4 shows the comparison of AVR's transient response for normalized uncertainty of amplifier and exciter. From Table 4, for small amounts of uncertainty in the amplifier and exciter, the nominal AVR system without a controller becomes unstable. In contrast, the AVR with FPA-PID, TLbO-PID, PIR-PID, and ZN-PID controllers becomes unstable when the normalized uncertainty in the amplifier and exciter is *x* = 1.5, *x* = 1.5, *x* = 1, and *x* = 3 correspondingly. With the V-Tiger PID controller, the permitted normalized uncertainty is measured as 3.5. This investigation makes it evident that the AVR with V-Tiger PID controller shows superior robustness performance than the other controllers.

**Table 4:** AVR's transient response for a range of normalized uncertainty of amplifier and exciter.

Normalized uncertainty	No controller			V-Tiger-PID			FPA-PID		
	T_r_ (s)	T_s_ (s)	%Os	T_r_ (s)	T_s_ (s)	%Os	T_r_ (s)	T_s_ (s)	%Os
*x* = 0	0.437	5.87	50.53	0.219	0.931	7.32	0.215	0.10	29.5
*x* = 0.5	Unstable			0.162	1.36	34.9	0.167	2.51	59.3
*x* = 1	Unstable			0.145	2.08	54.5	0.153	5.88	81
*x* = 1.5	Unstable			0.138	3.48	68.5	0.144	28.4	97
*x* = 2	Unstable			0.136	5.38	79.1	Unstable		
*x* = 2.5	Unstable			0.129	9.09	87.4	Unstable		
*x* = 3	Unstable			0.126	18.1	94	Unstable		
*x* = 3.5	Unstable			0.125	84.9	99.3	Unstable		

Normalized uncertainty	TLbO-PID			PIR-PID			ZN-PID		
	T_r_(sec)	T_s_(sec)	%Os	T_r_(sec)	T_s_(sec)	%Os	T_r_(sec)	T_s_(sec)	%Os
*x* = 0	0.226	1.49	30.4	0.202	2.28	51.2	0.236	3.04	50.30
*x* = 0.5	0.174	2.64	60	0.171	5.42	79.7	0.148	1.33	45
*x* = 1	0.159	6.18	81.8	0.155	256	103	0.135	2.36	64.5
*x* = 1.5	0.15	35.1	98.2	Unstable			0.128	3.78	78.5
*x* = 2	Unstable			Unstable			0.124	6.33	89
*x* = 2.5	Unstable			Unstable			0.121	12.9	97.1
*x* = 3	Unstable			Unstable			0.118	47.8	104
*x* = 3.5	Unstable			Unstable					

### Worst-case gain

**Figure 13:**
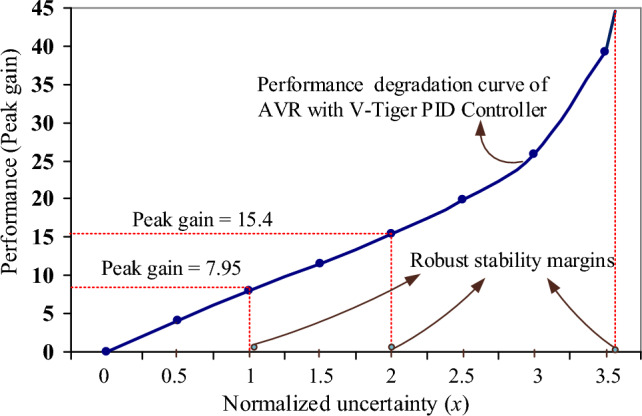
Performance degradation curve of AVR with V-Tiger PID controller.

The worst-case gain (K_wc_) is the peak gain at the specific uncertainty range. This value is the counterpart of the robust performance margin. Figure 13 shows the performance degradation curve of the AVR with the V-Tiger PID controller and it rises monotonically in proportion to the degree of uncertainty. The worst-case gain for the AVR with the V-Tiger-PID controller at the given uncertainty (*x* = 1) is around 7.95 dB, according to Fig. 13. When the specified uncertainty of the AVR using the V-Tiger-PID controller doubles (*x* = 2), the worst-cage gain hits 15.4 dB. Similarly, the AVR with FPA-PID, TLbO-PID, PIR-PID, and ZN-PID controllers has worst-case gains of about 15 dB, 15.1 dB, 47 dB, and 8.98 dB for the specified uncertainty. The AVR with FPA-PID, TLbO-PID, and PIR-PID controllers is unstable when the stated uncertainty is doubled; however, the AVR with ZN-PID controller has the worst-case gain of 17.6 dB.

## Conclusions and future research directions

In this study, a novel tuning technique known as Virtual Time response based iterative gain evaluation and re-design (V-Tiger) is proposed, and its use in adjusting PID controller gains for automated voltage regulator (AVR) systems is examined. The AVR system with V-Tiger tuning exhibits better transient response than other tuning techniques, as shown in Fig. 8 and Table 2. In particular, it performs better than the ZN, PIR, FPA, and TLbO PID controllers in terms of low peak overshoot, short settling time, and higher damping factor. Furthermore, by adding uncertainty to the field exciter and amplifier, the transient response of the AVR with the V-Tiger and ZN PID controllers is also shown.

The performance degradation curves of AVR with different controllers are plotted in Fig. 11b. From the curves, the robust stability margins and worst-case stability gains are measured. The robust stability margins of the AVR system using the ZN PID and V-Tiger PID controllers are 3 and 3.5, respectively, for the maximum allowable uncertainty of the amplifier and field exciter. Moreover, the worst-case stability gain with a ZN PID controller is 8.67 dB, and with a V-Tiger PID controller, it is 7.95 dB. Future research will use novel optimization tuning techniques, such as the honey badger algorithm and the dandelion optimizer, to examine the transient responsiveness of the generator output and the robustness of the AVR with the controller. Also, the proposed methodologies will be used for Automatic generation control, Power System Stabilizer, speed control of industrial DC drives, and distributed network voltage profile improvement.

Future research in this area shows potential for further advancements in voltage regulation and control strategies, with an emphasis on improving system performance and robustness. One possible avenue of investigation is the use of novel optimization techniques, such as the honey badger algorithm and the dandelion optimizer, to fine-tune control parameters and improve system responsiveness. Furthermore, there exists a potential to broaden the scope of the proposed approaches beyond the context of voltage regulation in power generation plants. Future research may look into the effectiveness of these technologies in alternative areas, including Automatic Generation Control (AGC), Power System Stabilization (PSS), and speed regulation of industrial DC drives. Moreover, research studies may explore the integration of these control strategies into distributed energy systems with the aim of improving voltage profile management and ensuring grid stability. Furthermore, the development of better predictive modeling tools may provide more precise forecasting of voltage variations, allowing for proactive mitigation strategies. Integration with machine learning algorithms could improve system adaptability and efficiency by allowing for real-time decision-making depending on changing grid conditions.

In conclusion, future research should focus on enhancing the current level of knowledge in voltage regulation and control, exploring applications that involve various disciplines, and addressing new challenges related to sustainability and resilience in power systems.

## Data Availability

The datasets used and/or analysed during the current study available from the corresponding author on reasonable request.

## References

[1] Li, K., Ji, L., Yang, S., Li, H. & Liao, X. Couple-group consensus of cooperative–competitive heterogeneous multiagent systems: A fully distributed event-triggered and pinning control method. *IEEE Trans. Cybern.* **52**, 4907–4915. 10.1109/TCYB.2020.3024551 (2022). 33055047 10.1109/TCYB.2020.3024551

[2] Shirkhani, M. *et al.* A review on microgrid decentralized energy/voltage control structures and methods. *Energy Rep.* **10**, 368–380. 10.1016/j.egyr.2023.06.022 (2023). 10.1016/j.egyr.2023.06.022

[3] Shen, Y., Liu, D., Liang, W. & Zhang, X. Current reconstruction of three-phase voltage source inverters considering current ripple. *IEEE Trans. Transp. Electrif.* **9**, 1416–1427. 10.1109/TTE.2022.3199431 (2023). 10.1109/TTE.2022.3199431

[4] Cao, X. *et al.* A method of human-like compliant assembly based on variable admittance control for space maintenance. *Cyborg. Bionic Syst.*10.34133/cbsystems.0046 (2023). 37681017 10.34133/cbsystems.0046 PMC10482162

[5] Agwa, A., Elsayed, S. & Ahmed, M. Design of optimal controllers for automatic voltage regulation using Archimedes optimizer. *Intell. Autom. Soft Comput.* **31**, 799–815. 10.32604/iasc.2022.019887 (2022). 10.32604/iasc.2022.019887

[6] Meng, S. *et al.* Observer design method for nonlinear generalized systems with nonlinear algebraic constraints with applications. *Automatica* **162**, 111512. 10.1016/j.automatica.2024.111512 (2024). 10.1016/j.automatica.2024.111512

[7] Wu, W., Zhu, H., Yu, S. & Shi, J. Stereo matching with fusing adaptive support weights. *IEEE Access* **7**, 61960–61974. 10.1109/ACCESS.2019.2916035 (2019). 10.1109/ACCESS.2019.2916035

[8] Hou, M., Zhao, Y. & Ge, X. Optimal scheduling of the plug-in electric vehicles aggregator energy and regulation services based on grid to vehicle. *Int. Trans. Electr. Energy Syst.* **27**, e2364. 10.1002/etep.2364 (2017). 10.1002/etep.2364

[9] Ziegler, J. G. & Nichols, N. B. Optimum settings for automatic controllers. *J. Dyn. Syst. Meas. Control* **115**, 220–222. 10.1115/1.2899060 (1993). 10.1115/1.2899060

[10] Li, B., Guan, T., Dai, L. & Duan, G.-R. Distributionally robust model predictive control with output feedback. *IEEE Trans. Autom. Control*10.1109/TAC.2023.3321375 (2024). 10.1109/TAC.2023.3321375

[11] Xu, A. *et al.* A fuzzy intelligent computing approach for energy/voltage control of microgrids. *J. Math.* **2023**, 1–11. 10.1155/2023/5289114 (2023). 10.1155/2023/5289114

[12] Bai, X., Xu, M., Li, Q. & Yu, L. Trajectory-battery integrated design and its application to orbital maneuvers with electric pump-fed engines. *Adv. Space Res.* **70**, 825–841. 10.1016/j.asr.2022.05.014 (2022). 10.1016/j.asr.2022.05.014

[13] Gopi, P. & Reddy, K. H. Design of PI speed controller for 3-Ph converter fed DC motor drive using symmetrical optimization. *J. Appl. Sci. Eng.*10.6180/jase.202112_24(6).0003 (2021). 10.6180/jase.202112_24(6).0003

[14] Yang, M., Wang, Y., Xiao, X. & Li, Y. A robust damping control for virtual synchronous generators based on energy reshaping. *IEEE Trans. Energy Convers.* **38**, 2146–2159. 10.1109/TEC.2023.3260244 (2023). 10.1109/TEC.2023.3260244

[15] Zhang, X., Pan, W., Scattolini, R., Yu, S. & Xu, X. Robust tube-based model predictive control with Koopman operators. *Automatica* **137**, 110114. 10.1016/j.automatica.2021.110114 (2022). 10.1016/j.automatica.2021.110114

[16] Salih, A. M., Humod, A. T. & Hasan, F. A. Optimum design for PID-ANN controller for automatic voltage regulator of synchronous generator. In *2019 4th Sci. Int. Conf. Najaf*, 74–79 (IEEE, 2019) 10.1109/SICN47020.2019.9019367.

[17] Song, J., Mingotti, A., Zhang, J., Peretto, L. & Wen, H. Accurate damping factor and frequency estimation for damped real-valued sinusoidal signals. *IEEE Trans. Instrum. Meas.* **71**, 1–4. 10.1109/TIM.2022.3220300 (2022). 10.1109/TIM.2022.3220300

[18] Zaidi, A., Basith, I. I. & Khan, V. Intelligent PID controller for automatic voltage regulation. *Electr. Electron. Eng.*10.5923/j.eee.20221201.01 (2022). 10.5923/j.eee.20221201.01

[19] Zhang, X., Wang, Y., Yuan, X., Shen, Y. & Lu, Z. Adaptive dynamic surface control with disturbance observers for battery/supercapacitor-based hybrid energy sources in electric vehicles. *IEEE Trans. Transp. Electrif.* **9**, 5165–5181. 10.1109/TTE.2022.3194034 (2023). 10.1109/TTE.2022.3194034

[20] Zhang, X., Wang, Z. & Lu, Z. Multi-objective load dispatch for microgrid with electric vehicles using modified gravitational search and particle swarm optimization algorithm. *Appl. Energy* **306**, 118018. 10.1016/j.apenergy.2021.118018 (2022). 10.1016/j.apenergy.2021.118018

[21] Ma, K. *et al.* Reliability-constrained throughput optimization of industrial wireless sensor networks with energy harvesting relay. *IEEE Internet Things J.* **8**, 13343–13354. 10.1109/JIOT.2021.3065966 (2021). 10.1109/JIOT.2021.3065966

[22] Li, X. & Sun, Y. Stock intelligent investment strategy based on support vector machine parameter optimization algorithm. *Neural Comput. Appl.* **32**, 1765–1775. 10.1007/s00521-019-04566-2 (2020). 10.1007/s00521-019-04566-2

[23] Zhang, H., Wu, H., Jin, H. & Li, H. High-dynamic and low-cost sensorless control method of high-speed brushless DC motor. *IEEE Trans. Ind. Inform.* **19**, 5576–5584. 10.1109/TII.2022.3196358 (2023). 10.1109/TII.2022.3196358

[24] Joseph, S. B., Dada, E. G., Abidemi, A., Oyewola, D. O. & Khammas, B. M. Metaheuristic algorithms for PID controller parameters tuning: Review, approaches and open problems. *Heliyon* **8**, e09399. 10.1016/j.heliyon.2022.e09399 (2022). 35600459 10.1016/j.heliyon.2022.e09399 PMC9120253

[25] Xu, B. & Guo, Y. A novel DVL calibration method based on robust invariant extended Kalman filter. *IEEE Trans. Veh. Technol.* **71**, 9422–9434. 10.1109/TVT.2022.3182017 (2022). 10.1109/TVT.2022.3182017

[26] Okou, F. A., Akhrif, O. & Dessaint, L.-A. Decentralized multivariable voltage and speed regulator for large-scale power systems with guarantee of stability and transient performance. *Int. J. Control* **78**, 1343–1358. 10.1080/00207170500345816 (2005). 10.1080/00207170500345816

[27] Wang, L., Zou, T., Cai, K. & Liu, Y. Rolling bearing fault diagnosis method based on improved residual shrinkage network. *J. Braz. Soc. Mech. Sci. Eng.* **46**, 172. 10.1007/s40430-024-04729-w (2024). 10.1007/s40430-024-04729-w

[28] Hou, X. *et al.* A space crawling robotic bio-paw (SCRBP) enabled by triboelectric sensors for surface identification. *Nano Energy* **105**, 108013. 10.1016/j.nanoen.2022.108013 (2023). 10.1016/j.nanoen.2022.108013

[29] Lu, Y., Tan, C., Ge, W., Zhao, Y. & Wang, G. Adaptive disturbance observer-based improved super-twisting sliding mode control for electromagnetic direct-drive pump. *Smart Mater. Struct.* **32**, 017001. 10.1088/1361-665X/aca84e (2023). 10.1088/1361-665X/aca84e

[30] Yu, J., Dong, X., Li, Q., Lu, J. & Ren, Z. Adaptive practical optimal time-varying formation tracking control for disturbed high-order multi-agent systems. *IEEE Trans. Circuits Syst. I Regul. Pap.* **69**, 2567–2578. 10.1109/TCSI.2022.3151464 (2022). 10.1109/TCSI.2022.3151464

[31] Chatterjee, S. & Mukherjee, V. PID controller for automatic voltage regulator using teaching–learning based optimization technique. *Int. J. Electr. Power Energy Syst.* **77**, 418–429. 10.1016/j.ijepes.2015.11.010 (2016). 10.1016/j.ijepes.2015.11.010

[32] Ekinci, S., Hekimoglu, B. & Kaya, S. Tuning of PID controller for AVR system using salp swarm algorithm. In *2018 Int. Conf. Artif. Intell. Data Process*, 1–6 (IEEE, 2018)10.1109/IDAP.2018.8620809.

[33] Sambariya, D. K. & Gupta, T. Optimal design of PID controller for an AVR system using flower pollination algorithm. *J. Autom. Control*10.12691/automation-6-1-1 (2018). 10.12691/automation-6-1-1

[34] Kushwah, B., Batool, S., Gill, A. & Singh, M. ANN and ANFIS techniques for automatic voltage regulation. In *2023 4th Int. Conf. Emerg. Technol.*, 1–8 (IEEE, 2023) 10.1109/INCET57972.2023.10170217.

[35] Lawal, M. J., Hussein, S. U., Saka, B., Abubakar, S. U. & Attah, I. S. Intelligent fuzzy-based automatic voltage regulator with hybrid optimization learning method. *Sci. Afr.* **19**, e01573. 10.1016/j.sciaf.2023.e01573 (2023). 10.1016/j.sciaf.2023.e01573

[36] Gopi, P. *et al.* Dynamic behavior and stability analysis of automatic voltage regulator with parameter uncertainty. *Int. Trans. Electr. Energy Syst.* **2023**, 1–13. 10.1155/2023/6662355 (2023). 10.1155/2023/6662355

[37] Pachauri, N. Water cycle algorithm-based PID controller for AVR. *COMPEL Int. J. Comput. Math. Electr. Electron. Eng.* **39**, 551–567. 10.1108/COMPEL-01-2020-0057 (2020). 10.1108/COMPEL-01-2020-0057

[38] Mahdavi, M., Alhelou, H. H., Gopi, P. & Hosseinzadeh, N. Importance of radiality constraints formulation in reconfiguration problems. *IEEE Syst. J.*10.1109/JSYST.2023.3283970 (2023). 10.1109/JSYST.2023.3283970

[39] Gopi, P. *et al.* Optimal placement of DG and minimization of power loss using naked mole rat algorithm. In *2023 Int. Conf. Technol. Policy Energy Electr. Power*, 35–40 (IEEE, 2023).10.1109/ICT-PEP60152.2023.10351150.

[40] Meddeb, A., Jmii, H., Amor, N. & Chebbi, S. Voltage stability enhancement using FACTS devices. In *2020 4th Int. Conf. Adv. Syst. Emergent Technol.*, 257–260 (IEEE, 2020)10.1109/IC_ASET49463.2020.9318220.

[41] Shah, S. O., Arshad, A. & Alam, S. Reactive power compensation utilizing FACTS devices. In *2021 Int. Conf. Emerg. Power Technol.*, 1–6 (IEEE, 2021)10.1109/ICEPT51706.2021.9435455.

[42] Goud, B. S. *et al.* AGC of multi area multi fuel system with water cycle algorithm based 3DOF-PID controller and integration of PEVs. In *2021 Int. Conf. Data Anal. Bus. Ind.*, 464–469 (IEEE, 2021) 10.1109/ICDABI53623.2021.9655899.

[43] Naga Sai Kalian, C., Bajaj, M., Kamel, S. & Jurado, F. Load frequency control of multi-area power system with integration of SMES and plug-in electric vehicles. In *2022 4th Glob. Power, Energy Commun. Conf.*, 349–54(IEEE, 2022)10.1109/GPECOM55404.2022.9815760.

[44] Bajaj, M. & Singh, A. K. An MCDM-based approach for ranking the voltage quality in the distribution power networks. In *2020 IEEE 7th Uttar Pradesh Sect. Int. Conf. Electr. Electron. Comput. Eng.*, 1–6 (IEEE, 2020) 10.1109/UPCON50219.2020.9376535.

[45] Sahri, Y. *et al.* Effectiveness analysis of twelve sectors of DTC based on a newly modified switching table implemented on a wind turbine DFIG system under variable wind velocity. *Ain Shams Eng. J.* **14**, 102221. 10.1016/j.asej.2023.102221 (2023). 10.1016/j.asej.2023.102221

[46] Sivapriya, A. *et al.* Real-time hardware-in-loop based open circuit fault diagnosis and fault tolerant control approach for cascaded multilevel inverter using artificial neural network. *Front. Energy Res.*10.3389/fenrg.2022.1083662 (2023). 10.3389/fenrg.2022.1083662

[47] Gopi, P., Mahdavi, M. & Alhelou, H. H. Robustness and stability analysis of automatic voltage regulator using disk-based stability analysis. *IEEE Open Access J. Power Energy* **10**, 689–700. 10.1109/OAJPE.2023.3344750 (2023). 10.1109/OAJPE.2023.3344750

[48] Pachauri, N. *et al.* A robust fractional-order control scheme for PV-penetrated grid-connected microgrid. *Mathematics* **11**, 1283. 10.3390/math11061283 (2023). 10.3390/math11061283

[49] Gopi, P., Srinivasan, S. & Krishnamoorthy, M. Disk margin based robust stability analysis of a DC motor drive. *Eng. Sci. Technol. Int. J.* **32**, 101074. 10.1016/j.jestch.2021.10.006 (2022). 10.1016/j.jestch.2021.10.006

[50] Kalyan, C. N. S. *et al.* Comparative performance assessment of different energy storage devices in combined LFC and AVR analysis of multi-area power system. *Energies* **15**, 629. 10.3390/en15020629 (2022). 10.3390/en15020629

[51] Kalyan, C. N. S. *et al.* Performance enhancement of combined LFC and AVR system with the integration of HVDC line. In *2023 IEEE IAS Glob. Conf. Renew. Energy Hydrog. Technol.*, 1–6 (IEEE, 2023)10.1109/GlobConHT56829.2023.10087546.

[52] Kalyan, C. N. S. *et al.* Enhancement in interconnected power system performance with 3DOFPID regulator and plug-in electric vehicles incorporation. In *2023 Int. Conf. Adv. Power, Signal, Inf. Technol.*, 353–358 (IEEE, 2023) 10.1109/APSIT58554.2023.10201781.

[53] Sai Kalyan, C. N. *et al.* Fruit fly optimization technique based regulator for LFC of conventional power system with the integration of plugin electric vehicles. In *2023 5th Int. Youth Conf. Radio Electron. Electr. Power Eng.,* 1–6 (IEEE, 2023) 10.1109/REEPE57272.2023.10086898.

[54] Sai Kalyan, C. N. *et al.* Revealing the significance of time delays on the performance of diverse source power systems under fruit fly optimization tuned 3DOFTID regulator. In *2023 5th Int. Youth Conf. Radio Electron. Electr. Power Eng.*, 1–6 (IEEE, 2023) 10.1109/REEPE57272.2023.10086832.

[55] Wang, W., Liang, J., Liu, M., Ding, L. & Zeng, H. Novel robust stability criteria for lur’e systems with time-varying delay. *Mathematics* **12**, 583. 10.3390/math12040583 (2024). 10.3390/math12040583

[56] Feng, J., Wang, W. & Zeng, H.-B. Integral sliding mode control for a class of nonlinear multi-agent systems with multiple time-varying delays. *IEEE Access* **12**, 10512–10520. 10.1109/ACCESS.2024.3354030 (2024). 10.1109/ACCESS.2024.3354030

[57] Zhang, X. *et al.* Secure routing strategy based on attribute-based trust access control in social-aware networks. *J. Signal Process Syst.*10.1007/s11265-023-01908-1 (2024). 10.1007/s11265-023-01908-1

[58] Mou, J. *et al.* A machine learning approach for energy-efficient intelligent transportation scheduling problem in a real-world dynamic circumstances. *IEEE Trans. Intell. Transp. Syst.* **24**, 15527–15539. 10.1109/TITS.2022.3183215 (2023). 10.1109/TITS.2022.3183215

[59] Song, F., Liu, Y., Shen, D., Li, L. & Tan, J. Learning control for motion coordination in wafer scanners: toward gain adaptation. *IEEE Trans. Ind. Electron* **69**, 13428–13438. 10.1109/TIE.2022.3142428 (2022). 10.1109/TIE.2022.3142428

[60] Chen, B., Hu, J., Zhao, Y. & Ghosh, B. K. Finite-time velocity-free rendezvous control of multiple AUV systems with intermittent communication. *IEEE Trans. Syst. Man Cybern. Syst.* **52**, 6618–6629. 10.1109/TSMC.2022.3148295 (2022). 10.1109/TSMC.2022.3148295

[61] Zhao, L., Qu, S., Xu, H., Wei, Z. & Zhang, C. Energy-efficient trajectory design for secure SWIPT systems assisted by UAV-IRS. *Veh. Commun.* **45**, 100725. 10.1016/j.vehcom.2023.100725 (2024). 10.1016/j.vehcom.2023.100725

[62] Gopi, P., Ramesh, M. & Lalitha, M. P. Evaluation of automatic voltage regulator’s pid controller coefficients using python. In *2021 IEEE Madras Sect. Conf.*, 1–7 (IEEE, 2021) 10.1109/MASCON51689.2021.9563458.

[63] Fei, M., Zhang, Z., Zhao, W., Zhang, P. & Xing, Z. Optimal power distribution control in modular power architecture using hydraulic free piston engines. *Appl. Energy* **358**, 122540. 10.1016/j.apenergy.2023.122540 (2024). 10.1016/j.apenergy.2023.122540

[64] Hu, J., Wu, Y., Li, T. & Ghosh, B. K. Consensus control of general linear multiagent systems with antagonistic interactions and communication noises. *IEEE Trans. Automat. Control* **64**, 2122–2127. 10.1109/TAC.2018.2872197 (2019). 10.1109/TAC.2018.2872197

[65] Lu, C., Gao, R., Yin, L. & Zhang, B. Human–robot collaborative scheduling in energy-efficient welding shop. *IEEE Trans. Ind. Inform.* **20**, 963–971. 10.1109/TII.2023.3271749 (2024). 10.1109/TII.2023.3271749

[66] Li, S., Zhao, X., Liang, W., Hossain, M. T. & Zhang, Z. A Fast and accurate calculation method of line breaking power flow based on Taylor expansion. *Front. Energy Res.*10.3389/fenrg.2022.943946 (2022). 10.3389/fenrg.2022.943946

[67] Bai, X., He, Y. & Xu, M. Low-thrust reconfiguration strategy and optimization for formation flying using jordan normal form. *IEEE Trans. Aerosp. Electron. Syst.* **57**, 3279–3295. 10.1109/TAES.2021.3074204 (2021). 10.1109/TAES.2021.3074204

[68] Zhang, J. *et al.* Fractional order complementary non-singular terminal sliding mode control of PMSM based on neural network. *Int. J. Automot. Technol.*10.1007/s12239-024-00015-9 (2024). 10.1007/s12239-024-00015-9

[69] Lu, C., Liu, Q., Zhang, B. & Yin, L. A Pareto-based hybrid iterated greedy algorithm for energy-efficient scheduling of distributed hybrid flowshop. *Expert Syst. Appl.* **204**, 117555. 10.1016/j.eswa.2022.117555 (2022). 10.1016/j.eswa.2022.117555

[70] Kosaka, M., Kosaka, A. & Kosaka, M. Virtual time-response based iterative gain evaluation and redesign. *IFAC-PapersOnLine* **53**, 3946–3952. 10.1016/j.ifacol.2020.12.2249 (2020). 10.1016/j.ifacol.2020.12.2249

